# Good clinical scores, no evidence of excessive anterior tibial translation, a high return to sport rate and a low re-injury rate is observed following anterior cruciate ligament reconstruction using autologous hamstrings augmented with suture tape

**DOI:** 10.1007/s00402-023-04835-9

**Published:** 2023-03-15

**Authors:** Jay R. Ebert, Peter Edwards, Peter T. Annear

**Affiliations:** 1 The School of Human Sciences (Exercise and Sport Science, M408), The University of Western Australia, 35 Stirling Highway, Crawley, WA 6009 Australia; 2 HFRC Rehabilitation Clinic, 117 Stirling Highway, Nedlands, WA 6009 Australia; 3 Perth Orthopaedic & Sports Medicine Research Institute, West Perth, WA 6005 Australia; 4 School of Allied Health, Curtin University, Perth, WA Australia; 5 Perth Orthopaedic and Sports Medicine Centre, West Perth, WA 6005 Australia

**Keywords:** Anterior cruciate ligament reconstruction, Augmentation, Clinical outcomes, Re-tears, Return to sport, Knee function

## Abstract

**Introduction:**

Augmented anterior cruciate ligament reconstruction (ACLR) techniques have been proposed to reduce the high reported re-injury rates and low rates of return to sport (RTS). This study reports clinical outcomes, RTS and re-injury rates in patients undergoing ACLR using autologous hamstrings augmented with suture tape.

**Materials and methods:**

A total of 53 patients were prospectively recruited, undergoing ACLR using hamstrings with suture tape augmentation, combined with a structured rehabilitation programme. Outcomes were collected to 24 months, including patient-reported outcome measures (PROMs), KT-1000 measurements, peak isokinetic knee strength and a four hop test battery. Limb Symmetry Indices (LSIs) were calculated for performance measures, whilst RTS rates, re-tears and re-operations were presented.

**Results:**

There were no significant side-to-side differences in anterior tibial translation between the operated and non-operated knees at 6 months (*p* = 0.433), with no increase (*p* = 0.841) in side-to-side anterior tibial translation from 6 to 24 months. At 24 months, 98.0% of patients demonstrated normal (< 3 mm) or near normal (3–5 mm) side-to-side differences. LSIs for peak knee extensor torque (*p* < 0.0001) and the single (*p* = 0.001), triple (*p* = 0.001) and triple crossover (*p* < 0.0001) hop tests for distance significantly improved. All PROMs significantly improved (*p* < 0.0001), with 70.2% and 85.7% of patients actively participating in pivoting sports at 12 and 24 months, respectively. Three patients underwent secondary procedures for meniscal symptoms. One patient suffered an ACL re-tear (17 months), with no further ipsilateral or contralateral injuries.

**Conclusion:**

ACLR with suture tape augmentation demonstrated no evidence of excessive anterior tibial translation, high-scoring PROMs, sound performance scores, a high rate of RTS and low re-injury rate.

## Introduction

Anterior cruciate ligament reconstruction (ACLR) is common [1] and, whilst a primary post-operative goal for many patients is a return to sport (RTS), it has been reported that across all patients, only 65% of patients return to their pre-injury level of sport [2]. Furthermore, an overall secondary re-injury rate of 7% has been reported, along with an 8% incidence of contralateral ACL tear, with a combined (ipsilateral and contralateral) ACL injury rate of 23% specifically in patients < 25 years of age who do RTS [3]. The reasons for re-injury are multifactorial [4], though a recent systematic review reported no significant differences in graft failure rates across varied graft types (quadriceps, hamstring and patellar tendon autografts, or allografts) [5]. In addition to ensuring that strength and functional performance is best restored given their link with re-injury risk [6, 7], surgical reconstruction techniques involving autograft (or allograft) augmentation have been proposed [8–13] in an attempt to improve outcomes and reduce re-injury rates. ACLR augmentation may permit early ACL reinforcement and graft stability prior to graft incorporation, also expediting post-operative recovery and accelerating rehabilitation [9, 14].

A range of augmented procedures and devices have been reported [15]. Encouraging clinical and RTS outcomes have been more recently reported when using a LARS ligament (LARS, Ligament Augmentation Reconstruction System, Corin Pty. Ltd.) to augment a hamstrings autograft [13, 16, 17], with patient outcomes of those undergoing augmented ACLR better than those undergoing non-augmented ACLR [16]. However, earlier use of synthetic augmentation, including LARS, appeared to present with excessive synovitis and in higher ACL graft failure rates [18–25]. A more recently employed device to augment an ACLR is FiberTape® (Arthrex, Naples, Florida, USA) [8, 12, 14, 26], with a retrospective comparison of outcomes in patients undergoing ACLR with and without suture augmentation with FiberTape® demonstrating improved outcomes with augmentation [14]. However, studies using FiberTape® augmentation are limited and a greater number of published papers exist related to the use of FiberTape® reinforcement in the context of ACL repair [27–29], rather than reconstruction, although even then many of these are technical notes and not studies reporting patient outcomes.

This study presents the clinical outcomes of a prospective patient cohort undergoing ACLR employing autologous hamstrings augmented with suture tape, combined with a progressive, structured rehabilitation programme. With the aforementioned reported re-injury and RTS rates in mind, it was hypothesized that: (1) no significant post-operative differences in anterior tibial translation would exist between the operated and non-operated limbs, (2) a low re-injury rate (< 5%) would be observed over the 24-month period, (3) a high RTS rate (> 70%) would be observed at 12 and 24 months and (4) a significant improvement in patient-reported outcome measures (PROMs) and objective outcomes would be observed following surgery.

## Material and methods

### Patients

**Fig. 1:**
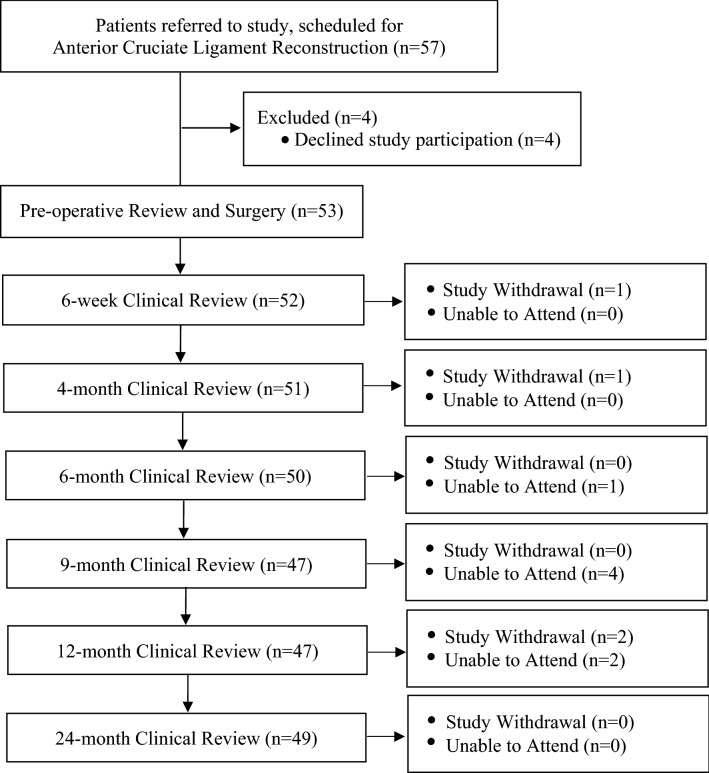
Flowchart demonstrating recruitment and evaluation over the post-operative period

Between March 2018 and November 2019, 57 patients scheduled for ACLR employing a hamstrings autograft and augmented with a suture tape were referred by a single surgeon in a private orthopaedic clinic for study discussion, recruitment and subsequent pre-operative review, of which 53 patients elected to participate (Fig. 1, Level IV prospective case series). Patients were candidates for surgery based on history, current symptoms and orthopaedic clinical examination, whilst magnetic resonance imaging (MRI) confirmed the ACL rupture in all patients. Patients were invited to participate in the study if they were deemed candidates for surgery, were 16–50 years of age (and skeletally mature) and required an isolated primary ACLR, with or without concomitant meniscal surgery. Whilst not encountered, patients were excluded from study participation if they presented with a body mass index (BMI) ≥ 40 or were unwilling or unable to participate in the post-operative rehabilitation protocol (outlined below). Ethics approval was provided by the relevant Human Research Ethics Committee (HREC) and the written consent of all participants was obtained prior to review.

### The surgical technique

All surgeries were performed by the senior author. Examination under anaesthesia was performed prior to tourniquet application to assess laxity of the injured ACL knee in comparison to the contralateral knee and clinically confirm a rupture of the ACL. Knee arthroscopy was subsequently performed to confirm the clinical diagnosis and further evaluate concomitant and/or chondral damage, which was addressed initially if required. Unstable ACL remnant tissue was then removed.

**Fig. 2:**
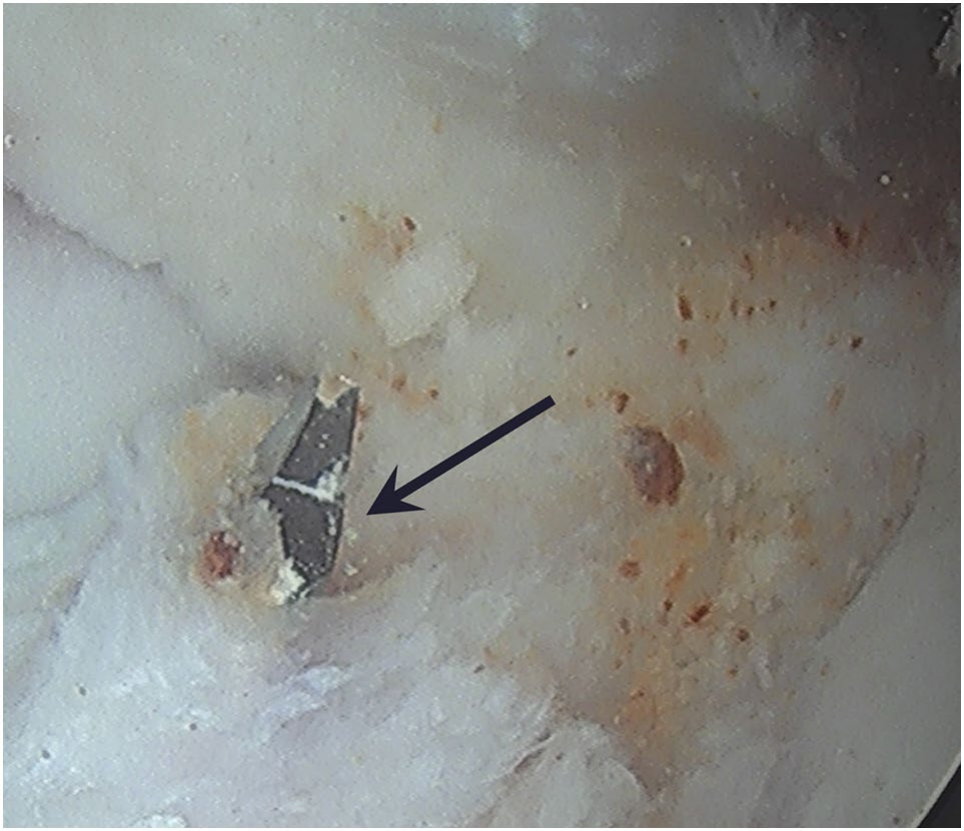
Tibial tunnel placement (left knee), shown existing the centre of the tibial ACL remnant (black arrow)

**Fig. 3:**
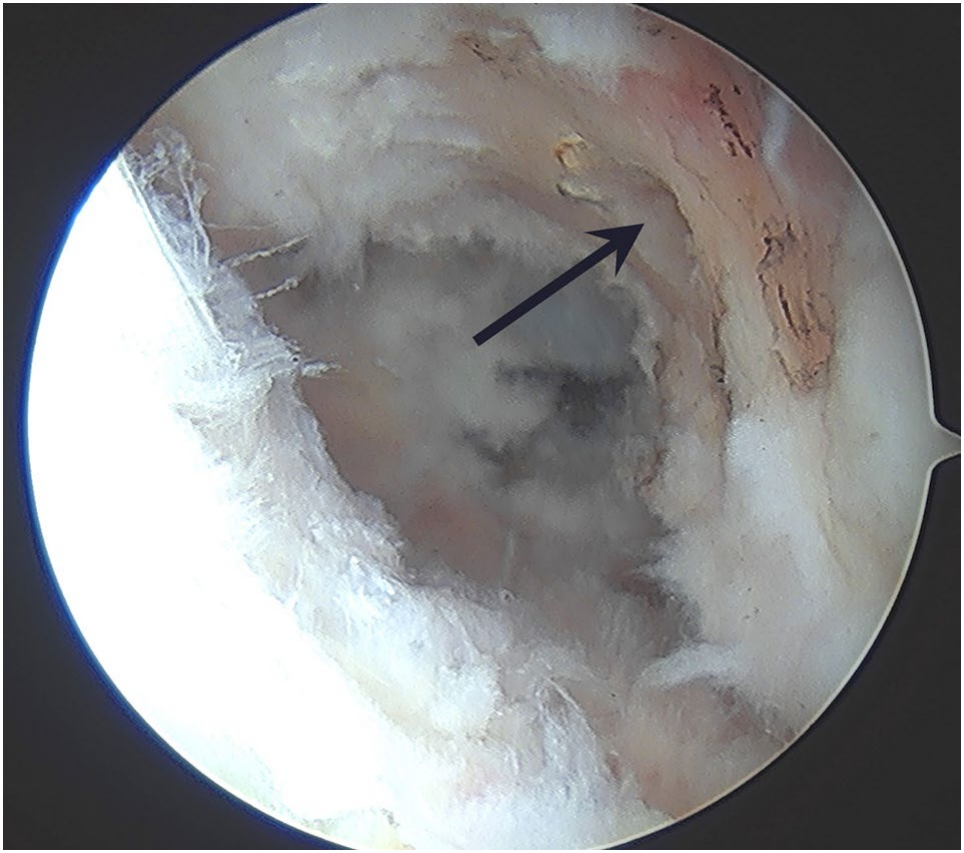
Femoral tunnel placement at 4 mm off the posterior femoral wall (black arrow), with tunnel position at the 2.00 o’clock position (left knee)

The ACL tunnels were routinely dictated by the anatomical positions of the existing ACL remnants. The tibial footprint of the ACL was initially identified, and all unstable remnant was removed. The tibial jig was placed centrally in the tibial footprint, and the tibial tunnel was prepared within the centre of the tibial ACL remnant (Fig. 2). Femoral tunnel preparation was performed in a similar way. The femoral anteromedial bundle soft tissue footprint was identified and an awl mark was created. A secondary check was via confirming a prepared tunnel position 2-4 mm off the posterior notch wall, generally in the 2.00 o’clock (left knee) or 10.00 o’clock (right knee) position (Fig. 3), with femoral tunnels drilled in maximal knee flexion. The ACL tibial remnant was cleared from the tibia to allow unobstructed passage of the graft within the knee.

**Fig. 4:**
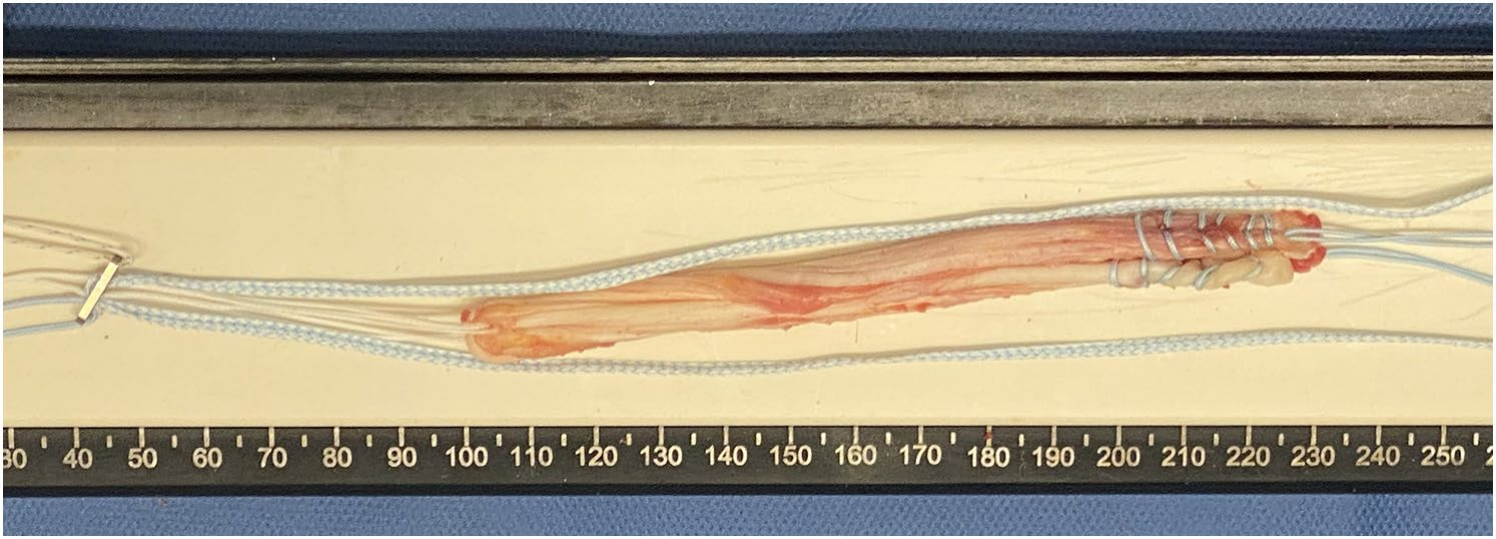
The graft construct, consisting of the semitendinosus and gracilis tendons and a FiberTape® (Arthrex, Naples, Florida, USA) acting as a ‘seat belt’ augmentation of the graft construct, creating a two-strand internal brace that was placed alongside the autograft

Semitendinosus and gracilis tendons were harvested from the ipsilateral knee through a 2–3 cm transverse incision approximately 1 cm above the pes anserinus, and prepared as doubled grafts. The combined diameter was measured to establish bone tunnel size reaming, with a minimum graft diameter of 8 mm confirmed for all cases. The harvested hamstring grafts were then passed through the ACL TightRope RT (Arthrex, Naples, Florida, USA) implant loop of the suspensory button creating a 4-strand hamstring graft. A FiberTape® (Arthrex, Naples, Florida, USA) was then attached by a half hitch to the femoral button to act as a ‘seat belt’ augmentation of the graft construct, creating a two-strand internal brace that was essentially placed alongside the autograft (Fig. 4).

**Fig. 5:**
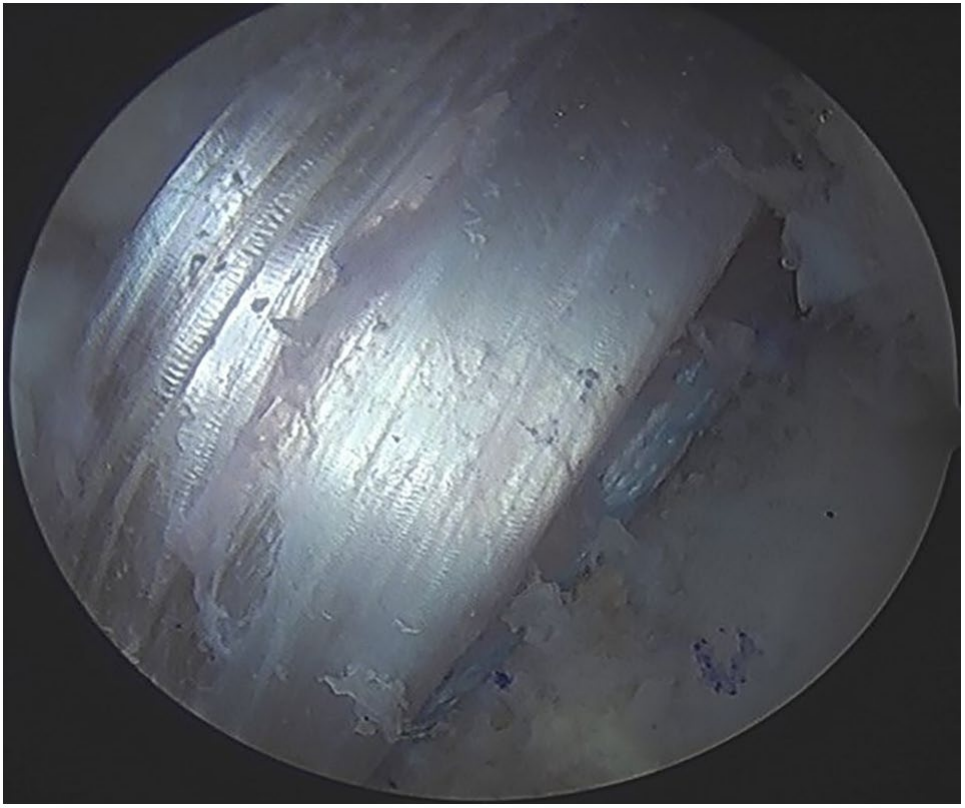
The graft construct, noting the FiberTape® acting as a ‘safety belt’

The graft was passaged after placing a suture via a shuttle technique from the tibia through to the button tunnel on the femur. The graft was seated with maximal manual tension whilst cycling the knee ten times. The tibial fixation was performed with a peek interference screw (Arthrex, Naples, Florida, USA), 1 mm larger than the tunnel and positioned in full knee extension. The two internal brace strands were fixed in an accessory position with a knotless anchor 1 cm distal to the tibial tunnel. The knee was place in full extension and the tight rope femoral suture was toggled to optimize maximum graft tension. The final graft construct is shown in Fig. 5.

### Rehabilitation

A standardized rehabilitation programme was implemented for all patients, aiming for a supervised therapist session every 2 weeks (starting from 2 weeks post-surgery) for the first 5–6 months (12 supervised sessions in total), with ongoing periodic review beyond 6 months post-surgery as required. These sessions were supplemented with an independent home and/or gym-based programme, aiming for 2–3 sessions in total per week. Whilst the home/gym-based programme was not closely monitored, 88.7% (47 of 53) of patients attended ≥ 75% of the designated supervised sessions, with the remaining 11.3% (6 of 53) of patients attending 58–67% of the designated sessions. This was generally due to geographical location and/or COVID-19 restrictions, and these patients were more closely monitored from afar as needed. All supervised rehabilitation was undertaken in a single, private out-patient therapy clinic. Table 1 provides an overview of the programme implemented. In brief, early post-operative management included weight bearing as tolerated, early circulatory (such as foot/ankle pumps) and knee range of motion (ROM) exercises, followed by a progressive programme aiming to restore strength and load capacity, with progression towards running and activities that better prepared the patient for an eventual RTS.

**Table 1:** Overview of the progressive rehabilitation programme undertaken by patients

Activity focus	Variable	Contents (and estimated timeframe)
Early protection, muscle activation and mobility	Pain/oedema	Cryotherapy and compression (day 1+)
	Knee bracing	Locked (1–2 weeks), no brace (week 2+^b^)
	Range of motion	Passive knee extension (day 1+)
		Passive and active-assisted knee flexion ROM (0–90°) (week 1–2), knee flexion ROM (0–120° +) (week 3+ ^a^)
	Weight bearing	Heel-toe gait as tolerated with 1–2 crutches (day 1+), Full/unaided weight bearing as tolerated (week 2+)
	Early muscle activation	Isometric quadriceps contractions (with electrostimulation) and resisted foot/ankle pumps (day 1+)
Exercises to restore strength and load capacity	Quadriceps and anterior hip dominant exercises	Isometric quadriceps contractions (with electrostimulation) (week 2+)
		Straight leg raises (upright and 45° external hip rotation) (week 2+)
		Isometric knee extensions (multi-angle at 90°, 60° and 45°) (Week 3 +)
		Isotonic knee extension (partial, 90–45°), unweighted (week 3+)
		Cycling—stationary (graduated low to high resistance as tolerated, and range permitting) (week 3+)
		Bilateral and unilateral seated leg press (week 3+)
		Wall/ball and free-standing squats (goblet, dumbbell) (week 3+)
		Lunges—stationary (week 3+), and walking and reverse slider disc (week 3+)
		Isotonic knee extension (full, 90–0°), unweighted (week 4+)
		Step ups / step downs (week 4–5)
		Single limb squats (ball/wall, free stand—week 5–7), squat variations (star excursion, Y-balance) (week 6–7)
	Hamstrings and posterior hip dominant exercises	Bridging (with and without TheraBand resistance) (week 2+)
		Standing hamstring curls (week 3+)
		Good-mornings (week 3+) and single-leg good-mornings (week 4+)
		Single limb bridging (week 4+)
		Ball bridge variations (single leg and hamstring curls) (week 4+)
		Seated knee flexion (hamstring curl)—unilateral (week 4+)
		Sliding leg curls: eccentric (week 4+), eccentric/concentric (week 8+) and single-leg (week 10+)
		Nordic curls (week 10+)
	Frontal plane hip stability and conditioning Concentric jump developments	Single leg balance and proprioceptive exercises (week 2+)
		Side-lying hip abduction and adduction (week 3+)
		Machine-based seated/standing hip abduction and adduction (week 3+)
		Resistance band side walks (week 4+)
	Ankle/calf exercises	Heel raises—bilateral (week 3+) and unilateral (week 4+)
Jump/landing preparation (plyometric)	Concentric jump developments	Vertical countermovement/squat jumps (week 9+)
		Horizontal broad jumps (week 9+) and box jumps (week 9+)
		Squat / broad jumps with single limb land (week 10+)
		Single limb hops (vertical and horizontal) (week 10+)
	Side-to-side jump integration	Side-to-side jumps over box (week 9+)
		Cross directional jumping exercises (± TheraBand) (week 10+)
		Cross directional unilateral bounding (± TheraBand) (week 10+)
	Continuous jumps	Continuous horizontal broad jumps (week 10+)
		Continuous horizontal hops (lateral/medial, triple, triple crossover) (week 12+)
	Advanced eccentric–concentric jump	Depth drops (double, single leg) from 20 cm, 30 cm and 40 cm (week 14+)
		Double (week 14+) and single (week 16+) leg drop jumps
Running and agility	Running	Trampoline jogging (week 8+)
		Jogging—flat surface, straight lines, intervals (week 10+)
		Running—flat surface, straight lines, intervals (week 13–14+)
	Agility	Running—backwards, lateral shuffle, grapevines (week 13–14+)
		Single limb hop variations (repeated, clock, square, speedy) (week 14+)
		Running—cross directional and controlled cutting manoeuvres (week 20+)
		Figure-8 runs, *t* test, Illinois etc. (week 22+)

^a^Modified for meniscal repair (0–90° for 6 weeks)

^b^Modified for meniscal repair (0–90° from 2–6 weeks)

Whilst late-stage progression through sport-specific training-based activities was also dependent on the patient’s specific sport, these aspects were not documented as part of the current patient cohort and patients transitioned through these components of training at their own discretion in collaboration with their sporting team. Whilst RTS was not advised until ≥ 9 months post-surgery and patients were counselled on specific objective criteria that should be attained before returning to sports activities (such as the restoration active knee extension ROM and flexion ROM LSI ≥ 90%, ≥ 90% LSI in hop tests and peak isokinetic knee extensor and flexor strength), this was not enforced and still largely at the final discretion of the patient.

### Patient assessment

First, all patients underwent a formal knee laxity exam performed in the clinic by the senior author (PA) at 4 months post-surgery, specifically to assess rotatory laxity grading via pivot shift evaluation. Anterior tibial translation (mm) was measured on both knees during a maximal manual test (MMT) using the KT-1000 knee arthrometer (MEDmetric Corp., San Diego, CA, USA) at 6, 9, 12 and 24 months post-surgery. Active knee flexion and extension range of motion (ROM, degrees) using a hand-held long-arm goniometer was assessed on the operated limb at 6 weeks, as well as 4, 6, 9, 12 and 24 months post-surgery. Patients underwent a 4-hop battery and assessment of peak isokinetic knee extensor and flexor strength (Nm) at 6, 9, 12 and 24 months. The 4-hop battery included the single hop for distance (SHD, m), the 6 m timed hop (6MTH, s), the triple hop for distance (THD, m) and the triple crossover hop for distance (TCHD, m) [30]. Peak isokinetic knee extensor and flexor strength was measured at 90°/s, using an isokinetic dynamometer (Isosport International, Gepps Cross, South Australia). These reviews and all nominated assessments (apart from the laxity exam undertaken by the senior author at 4 months) were undertaken by a qualified therapist, with 20 years of experience undertaking all of the aforementioned assessments.

Several patient-reported outcome measures (PROMs) were undertaken pre-surgery and at various post-operative time-points. These included the International Knee Documentation Committee (IKDC) Subjective Knee Evaluation Form [31], the Knee Outcome Survey (KOS) Activities of Daily Living Scale [32], the Cincinnati Knee Rating System (CKRS) [33], the Lysholm Knee Score (LKS) [34], the Tegner Activity Scale (TAS) [35], the Anterior Cruciate Ligament Return to Sport after Injury (ACL-RSI) [36] and the Noyes Sports Activity Rating Scale (NSARS) [37]. A satisfaction score was employed at 24 months post-surgery, evaluating patient satisfaction with the surgery overall, as well as with the surgery to relieve pain, improve the ability to perform normal daily and work activities, improve the ability to return to recreational activities (including walking, swimming, cycling, golf, dancing), and improve the ability to participate in sport (including sports such as tennis, netball, soccer and football). A Likert Response Scale was employed with descriptors Very Satisfied, Somewhat Satisfied, Somewhat Dissatisfied and Very Dissatisfied.

### Data and statistical analysis

For this prospective study, a priori sample size power calculation was determined based on the recommendations of Cohen [38] and employing data previously collected and published in patients undergoing ACLR with a hamstrings autograft, augmented with LARS [13]. Therefore, in using this existing data and for an anticipated moderate effect size (*d* = 0.67) in the primary outcome (anterior tibial translation as evaluated via side-to-side difference in anterior tibial translation in mm for the KT-1000 at 6 months post-surgery), assuming an SD of 3 mm and at alpha level of 0.05 and a power of 0.9, the sample size was estimated at 49 patients to demonstrate a significant difference in anterior tibial translation between the operated and non-operated knees. Overall, 53 patients were recruited to allow for attrition over the assessment period.

For all subjective (PROMs) and objective outcomes, the means (SD, range) were presented at the designated assessment time-points, whilst repeated-measures analysis of variance (ANOVA) was employed to assess change in these outcomes over time. Limb Symmetry Indices (LSIs) were calculated and presented for the hop and strength tests, further categorized by the number and percentage of patients with LSIs ≥ 90% for all four hop tests (at each time-point), as well as all hop tests combined with peak isokinetic knee extension and flexion torque. For KT-1000 anterior tibial translation measures, *t* tests were employed to compare the operated and non-operated limbs at 6 months post-surgery, whilst repeated-measures ANOVA assessed any change in the side-side limb anterior tibial translation difference over time. KT-1000 anterior tibial translation measures were further categorized based on side-to-side difference as normal (< 3 mm), nearly normal (3–5 mm), abnormal (6–10 mm) and severely abnormal (> 10 mm) [39]. The NSARS was employed to present the number (and percentage) of patients participating in Level 1 (participation 4–7 days/week) or Level 2 (participation 1–3 days per week) activities that included jumping, hard pivoting and cutting sports pre-injury and at 12- and 24 months post-surgery. The number (and percentage) of patients reporting ‘Very Satisfied’, ‘Somewhat Satisfied’, ‘Somewhat Dissatisfied’ and ‘Very Dissatisfied’ within each of the satisfaction domains at 24 months post-surgery was presented. The number (and type) of surgical complications, adverse events, re-operations and re-injuries were presented. Where appropriate, statistical analysis was performed using SPSS software (SPSS, Version 27.0, SPSS Inc., USA), with statistical significance determined at *p* < 0.05.

## Results

Patient demographics and injury/surgery parameters of the 53 patients that were recruited and underwent surgery are demonstrated in Table 2.

**Table 2:** Pre-operative patient demographics and injury/surgery parameters for the cohort that were consented and underwent pre-operative review

Variable	Measure	*n* = 53
Age (y)	Mean (SD)	28.1 (9.2)
	Range	16–45
Body mass index	Mean (SD)	24.9 (3.4)
	Range	18.8–39.8
Time injury to surgery (weeks)	Mean (SD)	9.1 (13.0)
	Range	2–52
Gender (males)	*n* (%)	30 (56.6)
Injury mechanism (non-contact)	*n* (%)	45 (84.9)
Graft diameter (mm)	Mean (SD)	8.5 (0.3)
	Range	8–9
Concomitant surgery	*n* (%)	26 (49.0)
Meniscectomy	*n* (%)	7 (13.2)
Meniscus repair	*n* (%)	19 (35.8)

### Objective results

With respect to the 4-month knee laxity exam undertaken by the senior author, all patients presented with a normal (or near normal) pivot shift clinical examination, with no Grade II or III pivot laxity outcomes. For the later-stage KT-1000 assessments, there were no significant anterior tibial translation differences between the operated and non-operated knees at 6 months post-surgery (*p* = 0.433), with no significant increase (*p* = 0.841) in side-to-side anterior tibial translation from 6 to 24 months (Table 3). At 24 months, KT-1000 measurements demonstrated normal (< 3 mm) or near normal (3–5 mm) side-to-side differences in 98.0% of patients (Table 3). Knee flexion and extension ROM significantly improved (*p* < 0.0001) over time, as did the LSI for peak isokinetic knee extensor torque (*p* < 0.0001), the SHD (*p* = 0.001), THD (*p* = 0.001) and TCHD (*p* < 0.0001) (Table 4). At 12 months post-surgery, 72.3% of patients presented with an LSI ≥ 90% for every hop test, which dropped to 53.2% of patients when combined with LSIs ≥ 90% for peak isokinetic knee extensor and flexor strength (Table 5). This was 79.6% of patients (all four hops) and 61.2% of patients (all four hops combined with strength measures) at 24 months post-surgery (Table 5).

**Table 3:** KT-1000 knee arthrometer side-to-side anterior tibial translation difference (mm) scores at 6, 9, 12 and 24 months post-surgery

Variable	Measure	6 months (*n* = 50)	9 months (*n* = 47)	12 months (*n* = 47)	24 months (*n* = 49)	Time effect (*p* value)
KT-1000, side-to-side difference (mm)	Mean (SD), range	1.1 (1.0), 0–5	1.0 (0.9), 0–5	1.0 (0.9), 0–5	1.0 (1.0), 0–5	0.841
Normal (< 3 mm)	*n* (%)	48 (96.0)	45 (95.7)	45 (95.7)	46 (93.9)	N/A
Nearly normal (3–5 mm)	*n* (%)	2 (4.0)	2 (4.3)	2 (4.3)	2 (4.1)	N/A
Abnormal (6–10 mm)	*n* (%)	0 (0.0)	0 (0.0)	0 (0.0)	1 (2.0)	N/A
Severely abnormal (> 10 mm)	*n* (%)	0 (0.0)	0 (0.0)	0 (0.0)	0 (0.0)	N/A

The number (and percentage) of knees graded as normal (< 3 mm), nearly normal (3–5 mm), abnormal (6–10 mm) or severely abnormal (> 10 mm) in each group is shown. *p* value represents the change in the side-to-side limb difference over time

Note: 24-month review includes the patient that suffered an ACL re-tear at 17 months post-surgery

**Table 4:** Knee flexion and extension range of motion (degrees, operated limb) along with Limb Symmetry Indices (LSIs) for peak isokinetic knee extensor and flexor torque and the four single hop tests. Shown are means (SD)

Time-point	Knee flexion (degrees)	Knee extension (degrees)	Knee extensor torque LSI	Knee flexor torque LSI	SHD LSI	6MTH LSI	THD LSI	TCHD LSI
6 weeks	128.1 (10.7)	1.8 (2.2)	N/A	N/A	N/A	N/A	N/A	N/A
4 months	135.8 (8.5)	0.5 (1.4)	N/A	N/A	N/A	N/A	N/A	N/A
6 months	138.8 (7.9)	0.5 (1.8)	75.1 (17.9)	92.8 (13.9)	91.9 (7.7)	93.3 (10.0)	91.1 (9.2)	90.0 (8.6)
9 months	141.3 (8.1)	0.3 (2.0)	82.2 (19.5)	97.7 (13.6)	93.7 (8.2)	96.7 (8.2)	93.8 (7.2)	93.7 (8.0)
12 months	142.2 (7.9)	-0.1 (2.0)	92.7 (10.0)	98.3 (13.6)	96.0 (6.9)	96.2 (5.8)	95.8 (7.5)	96.1 (6.8)
24 months	144.5 (7.0)	-0.8 (2.1)	95.2 (10.8)	97.7 (9.3)	97.7 (5.0)	96.5 (7.3)	97.6 (5.1)	96.8 (6.2)
*p* value	< 0.0001	< 0.0001	< 0.0001	0.114	0.001	0.179	0.001	< 0.0001

SHD single hop for distance, 6MTH 6 m timed hop, THD triple hop for distance, TCHD triple crossover hop for distance

**Table 5:** The number (and percentage) of patients at each time-point that had Limb Symmetry Indices (LSIs) ≥ 90% for every one of the four hop tests employed, as well as every one of the four hop tests combined with peak isokinetic knee extensor and flexor torque

Time-point	≥ 90% (all 4 × hops)	≥ 90% (all 4 × hops and peak knee extensor and flexor torque)
6 months (*n* = 50)	22 (44.0)	9 (18.0)
9 months (*n* = 47)	32 (68.1)	17 (36.2)
12 months (*n* = 47)	34 (72.3)	25 (53.2)
24 months (*n* = 49)	39 (79.6)	30 (61.2)

### Subjective results and return to sport

All PROMs significantly improved over time (*p* < 0.0001) (Table 6). As per the NSARS, 90.6% of patients were actively participating in Level 1 or 2 sports that included jumping, hard pivoting, cutting, running, twisting and/or turning pre-injury, which was 70.2% and 85.7% at 12 and 24 months post-surgery, respectively (Table 7). At 24-month review, 98.0% of patients were satisfied overall with their surgical outcome, with 93.9% satisfied with their ability to participate in sport (Table 8).

**Table 6:** Patient-reported outcome measures (PROMs) throughout the pre- and post-operative timeline. Shown are means (SD)

Time-point	IKDC	ACL-RSI	KOS	Cincinnati	Lysholm	Tegner
Pre-surgery	48.0 (17.5)	N/A	52.0 (14.0)	49.5 (19.0)	54.8 (20.0)	7.0 (1.5)
6 weeks	55.8 (13.7)	N/A	62.4 (10.5)	63.6 (16.7)	74.5 (16.2)	3.3 (0.9)
4 months	72.2 (9.7)	N/A	70.0 (6.7)	76.8 (11.9)	84.9 (9.9)	4.2 (0.9)
6 months	79.2 (9.4)	49.1 (19.7)	72.9 (5.2)	82.5 (8.5)	87.2 (8.4)	4.9 (1.1)
9 months	82.6 (9.7)	60. (20.9)	N/A	N/A	N/A	N/A
12 months	88.9 (8.9)	66.4 (21.3)	76.3 (3.5)	91.8 (7.3)	93.8 (5.9)	6.4 (1.5)
24 months	94.0 (6.4)	73.7 (19.7)	78.0 (4.1)	96.2 (5.2)	96.5 (4.6)	7.1 (1.5)
Time effect (*p* value)	< 0.0001	< 0.0001	< 0.0001	< 0.0001	< 0.0001	< 0.0001

IKDC International Knee Documentation Committee Subjective Knee Evaluation Form, ACL-RSI Anterior Cruciate Ligament Return to Sport after Injury Score, KOS Knee Outcome Survey

Note: the pre-surgery Tegner represents the pre-injury score (ANOVA analysis only includes post-surgery values for the Tegner)

**Table 7:** The percentage of patients pre-surgery, as well as 12 and 24 months post-surgery, actively participating in Level 1 (participation 4–7 days/week) or Level 2 (participation 1–3 days per week) Noyes activities that included jumping, hard pivoting, cutting, running, twisting and/or turning sports

Time-point	*n* (%)
Pre-surgery (*n* = 53)	48 (90.6)
12 months (*n* = 47)	33 (70.2)
24 months (*n* = 49)	42 (85.7)

Note: 24-month review includes the patient that suffered an ACL re-tear at 18 months post-surgery

**Table 8:** The number of patients at 24 months post-surgery (*n* = 49) within each of the four satisfaction gradings (very Satisfied, somewhat satisfied, somewhat dissatisfied, very dissatisfied) for each of the five satisfaction items

Satisfaction item	Pain relief	Improving ability to undertake ADLs	Improving ability to participate in recreational activities	Improving ability to participate in sport	Overall satisfaction
Very satisfied	42	44	42	36	41
Satisfied	5	5	6	10	7
Dissatisfied	2	0	1	3	1
Very dissatisfied	0	0	0	0	0
Satisfied overall, *n* (%)	47 (95.9)	49 (100.0)	48 (98.0)	46 (93.9)	48 (98.0)

### Complications, re-injuries and secondary surgical procedures

Over the course of the 24-month follow-up period, one patient presented with an early wound infection that was treated accordingly without further issue. Three patients underwent secondary surgical procedures, including one patient that underwent arthroscopic lateral meniscectomy for recurrent symptoms at 18 months after his primary ACLR (with an intact ACL at time of secondary surgery) and one patient that underwent lateral meniscal repair at 10 months after his primary ACLR (with an intact ACL at time of secondary surgery, albeit the meniscal tear was new and following a secondary incident). The third patient underwent medial meniscectomy at 6 months after his primary ACLR for recurrent symptoms and, whilst he was doing well and had returned to pivoting sports by 12 months, experienced an ACL re-tear at 17 months after his primary ACLR which continues to be managed non-operatively. This patient had a graft diameter of 9 mm. There were no further ipsilateral re-tears or contralateral tears. The data collected from these patients were still included in the results analysis.

## Discussion

The most important finding from the current study was that an ACLR technique using autologous hamstrings augmented with a suture tape, combined with a structured post-operative rehabilitation programme, produced high-scoring PROMs and patient satisfaction with encouraging performance scores and RTS rates, without evidence of excessive anterior tibial translation and/or a high re-injury rate.

No difference in anterior tibial translation between the operated and non-operated limbs was observed, with 98% of patients demonstrating normal (< 3 mm) or near normal (3–5 mm) side-to-side differences up until 24 months post-surgery (the only patient who demonstrated side-to-side anterior tibial translation > 5 mm had suffered a known re-tear). This was in support of the first hypothesis. Further to this, as reported recently by Fiil et al. [40], excessive post-operative anterior tibial translation may be associated with worse knee-related quality of life, reduced function in sports and an increased revision rate. Whilst the rationale for graft augmentation is largely focussed on early graft reinforcement [9, 14], the true nature of this reinforcement capacity remains unknown, given the relative lack of biomechanical research on suture tape augmentation. A biomechanical study published by Massey et al. [41] reported a higher load to failure, stiffness and energy to failure when augmenting a graft with internal brace, though this was in the context of ACL repair (not reconstruction). In the current study, only one patient (2%) suffered an ACL re-injury with no contralateral ACL tears up until 24 months, also in support of the second hypothesis. However, it should be acknowledged that whilst Grindem et al. [6] reported an increased re-tear rate up until 9 months post-surgery after which time no further reduction in re-tear risk was observed, theoretically an elevated re-tear risk may extend well after the patient’s RTS so ongoing review is required. Whilst excessive synovitis and high failure rates had limited the ongoing early use of synthetics in ACLR [18–25], these complications were not observed in the current study.

In the current study, 70.2% of patients were actively participating in pivoting sports at 12 months post-surgery, which had increased to 85.7% at 24 months (noting that 90.6% of patients were actively participating in pivoting sports pre-injury). This supported the third hypothesis and, of further interest, the 24-month post-operative mean TAS was actually higher than the pre-injury TAS. Whilst similar RTS rates were previously reported in patients following ACLR augmented with LARS [13], Ardern et al. [2] reported that only 65% of patients return to their pre-injury level of sport, with 55% returning to competitive sport. The higher RTS rates may be influenced by a range of factors including participation and ongoing progression of rehabilitation, which was well adhered to in the current study. Further to this, the underlying rationale for the use of ACLR augmentation is that it may permit early ACL reinforcement and graft stability prior to graft incorporation, also accelerating rehabilitation [9, 14]. Of importance, the encouraging RTS rates currently observed did not appear to increase the risk of excessive anterior tibial translation or re-injury risk. It should be reiterated again that RTS was not advised until ≥ 9 months post-surgery and patients were counselled on specific objective criteria that should be ideally attained before RTS, though this could not be enforced and was at the final discretion of the patient.

High-scoring PROMs and high levels of patient satisfaction were reported, whilst mean LSIs ≥ 90% were reported at all post-operative time-points for peak isokinetic knee flexor strength and all hop measures. Furthermore, the mean LSI for peak isokinetic knee extensor strength was ≥ 90% at 12 and 24 months, albeit 75% and 82% at 6 and 9 months, respectively. This was largely in support of the fourth hypothesis. However, when grouped in the form of a performance test battery, 72% and 80% of patients presented with an LSI ≥ 90% for every hop test at 12 and 24 months, respectively. When this test battery further included LSIs ≥ 90% for the knee extensor and flexor strength measures, this was only 53% and 61% at 12 and 24 months, respectively. Despite the low re-injury rate currently observed, existing research has reported an increased re-injury risk if patients fail to meet LSIs ≥ 90% across a range of tests including strength and hop performance measures [6, 7]. In contrast, other research has suggested an increased risk of contralateral ACL injury in the presence of improved strength and/or hop performance symmetry [42, 43]. Therefore, the limitations of employing LSIs to present performance outcomes should be acknowledged, such as the variation in LSI ‘cut-off’ values employed [6, 44–47] and the potential for LSIs to overestimate function [48].

Whilst the current subjective, objective and RTS outcomes appear similar to those reported previously in patients undergoing ACLR augmented with LARS [13], and more recent longer term follow-ups of reconstruction/repair with and without other ligament augmentation devices have reported sound clinical results [49, 50], limited published outcomes exist presenting outcomes specifically after ACLR augmented with FiberTape®. Bodendorfer et al. [14] presented a retrospective comparison of outcomes in patients undergoing ACLR with and without FiberTape® suture augmentation, with augmentation demonstrating less pain, improved PROMs and improved early return to activity, without evidence of over-constraint. A retrospective cohort study published by Barnas et al. [51] reported comparable functional outcomes in patients undergoing surgery for partial ACL tears with synthetic augmentation using either a polyethylene terephthalate tape (Neoligaments) or FiberTape® suture augmentation. A recent retrospective comparison published by Hopper et al. [52] reported comparable re-injury and secondary surgery rates in patients undergoing ACLR versus those undergoing ACL repair with suture tape augmentation, in the context of acute proximal ACL ruptures. Finally, a recent systematic review published by Zheng et al. [53] specifically on the use of suture augmentation for ACLR reported overall favourable clinical outcomes and, whilst being associated with better sports performance compared to standard ACLR, was comparable in most functional scores, knee stability measures and graft failure rates. Most other ACLR papers employing FiberTape® augmentation are technical notes without patient outcomes [8, 12, 26]. A prospective 2-year study published by Heusdens et al. [27] reported improved post-operative outcomes of suture augmentation in the context of ACL repair, with a 4.8% re-rupture rate over the period, but other published papers using FiberTape® augmentation for ACL repair are also limited to technical notes [28, 29].

A number of limitations are acknowledged within the current study. First, it was a single centre study in patients undergoing a specific augmented ACLR technique that does not permit generalization. Furthermore, we acknowledge that there was no comparative group with the current study and, based on the early clinical experience our group had with this augmented ACLR technique, our initial plan was to undertake a robust prospective evaluation of patients undergoing this ACLR technique with close and frequent assessment of outcomes and adverse events, with comparison to existing literature where appropriate. This now provides a framework for a subsequent randomized comparative study. Additionally, it may be argued that it was a heterogeneous group with a wide age range (16–45 years) and almost 50% of patients undergoing concomitant meniscal surgery, though this is also a strength in presenting outcomes in a common community-level cohort embarking on ACLR. Second, we acknowledge that the primary study aim and sample size calculation was focussed around excessive anterior tibial translation (KT-1000 measurements), and both the 4-month pivot shift clinical review, as well as the 6-, 9-, 12- and 24-month KT-1000 reviews, were undertaken on the patient (on both limbs for the KT-1000) in an awake condition, which may be less reliable than an anaesthetized environment. Third, whilst an aim was to report on RTS rates at 12 and 24 months, the actual time to RTS was not documented. Finally, whilst it is acknowledged that rehabilitation can affect strength and function after ACLR [45, 54, 55] and patients underwent a structured rehabilitation programme following surgery (also seeking to document rehabilitation adherence), it is acknowledged that in many community-level ACLR patients, rehabilitation will differ, as will individual patient motivation and exercise diligence.

## Conclusion

The current study has demonstrated that ACLR using autologous hamstrings augmented with the suture tape, combined with a structured, post-operative rehabilitation programme, produced high-scoring PROMs and patient satisfaction with encouraging performance scores and RTS rates, without evidence of excessive anterior tibial translation and/or a high re-injury rate. Particularly given the high RTS rates at 24 months post-surgery, ongoing patient review is required to further investigate latter stage re-injury rates.

## Data Availability

The data that support the findings of this study are available from the corresponding author, upon reasonable request.
